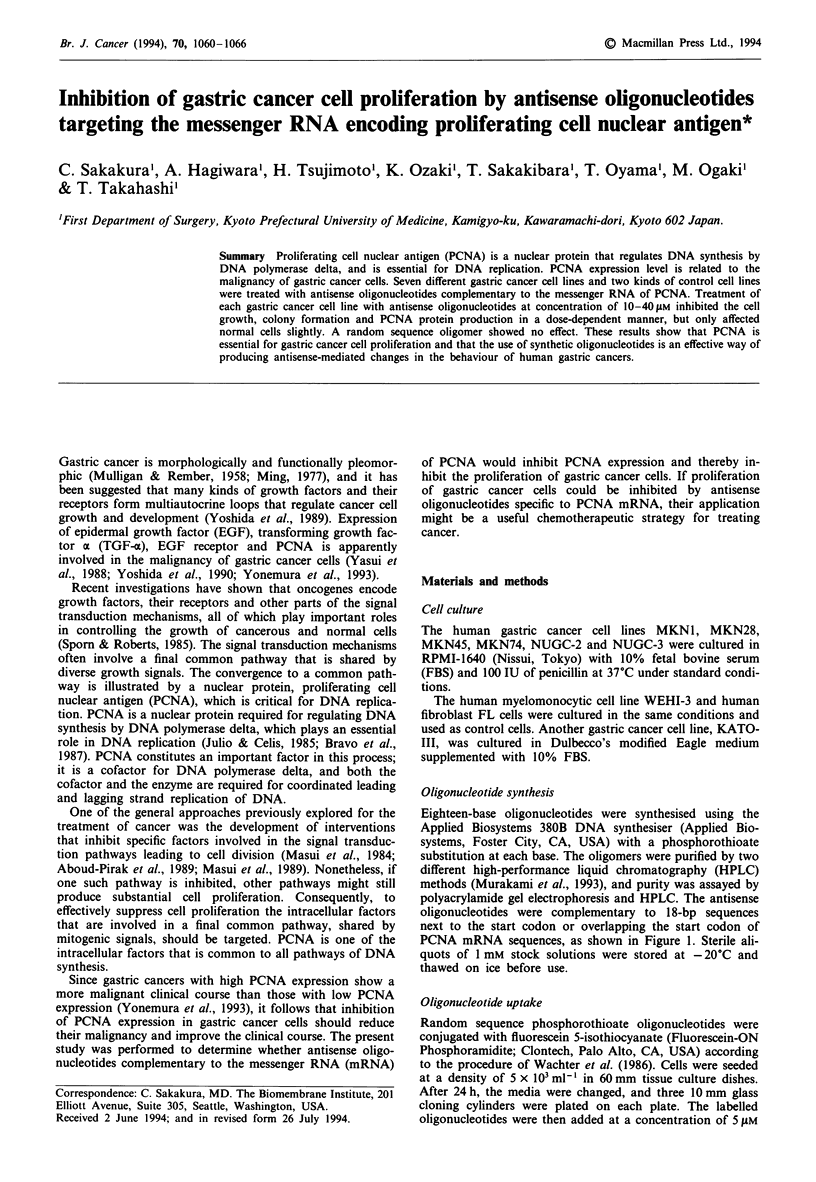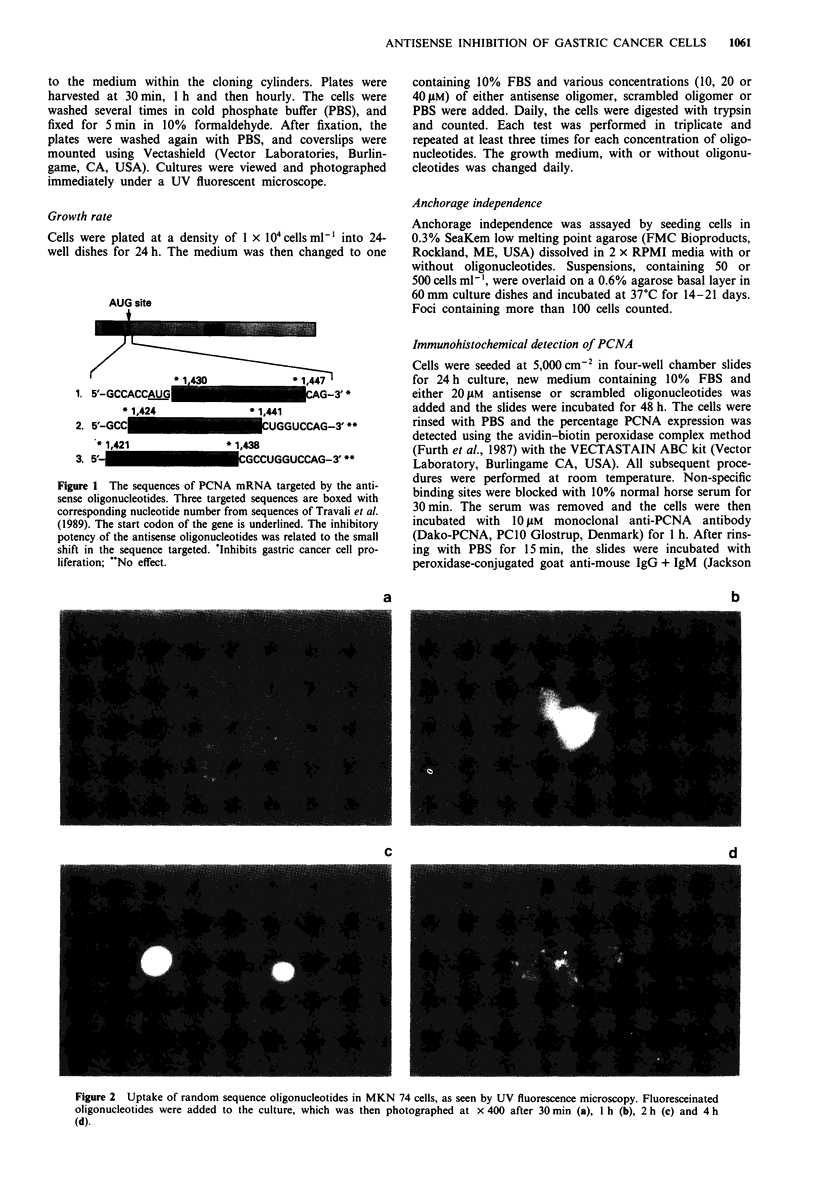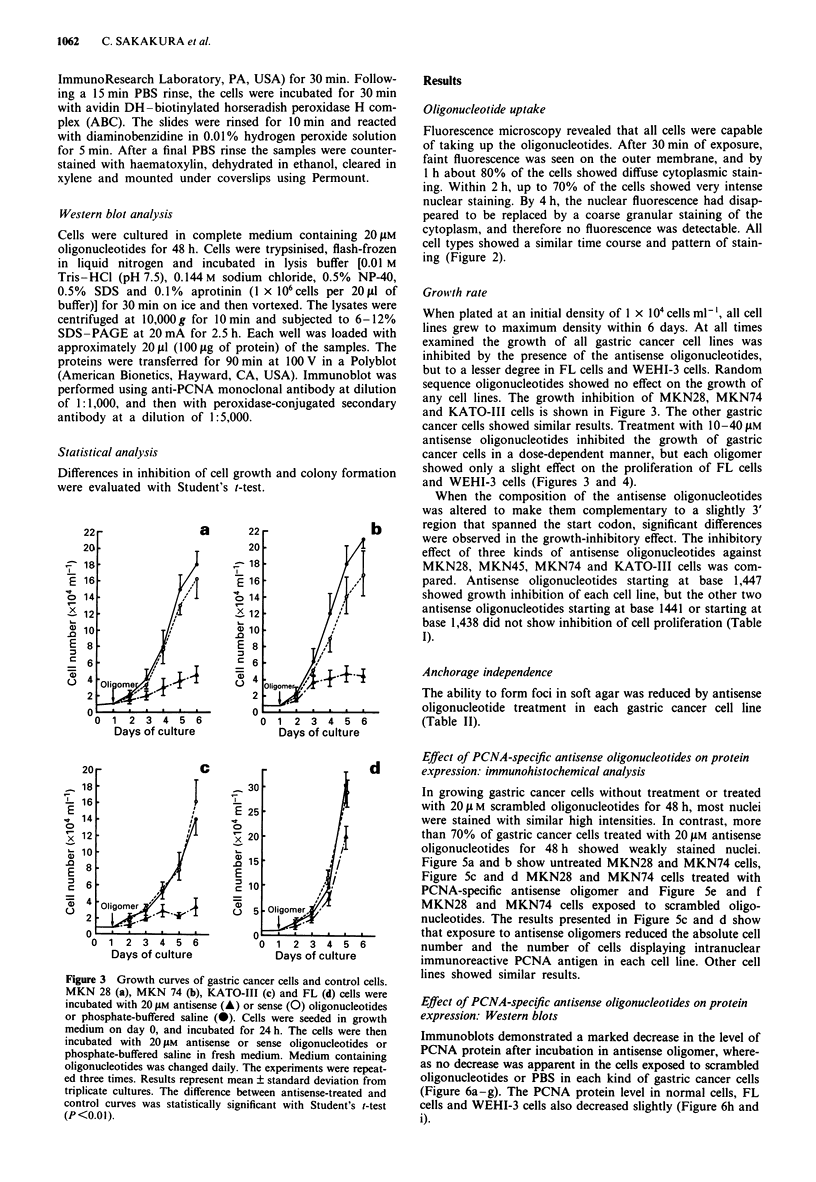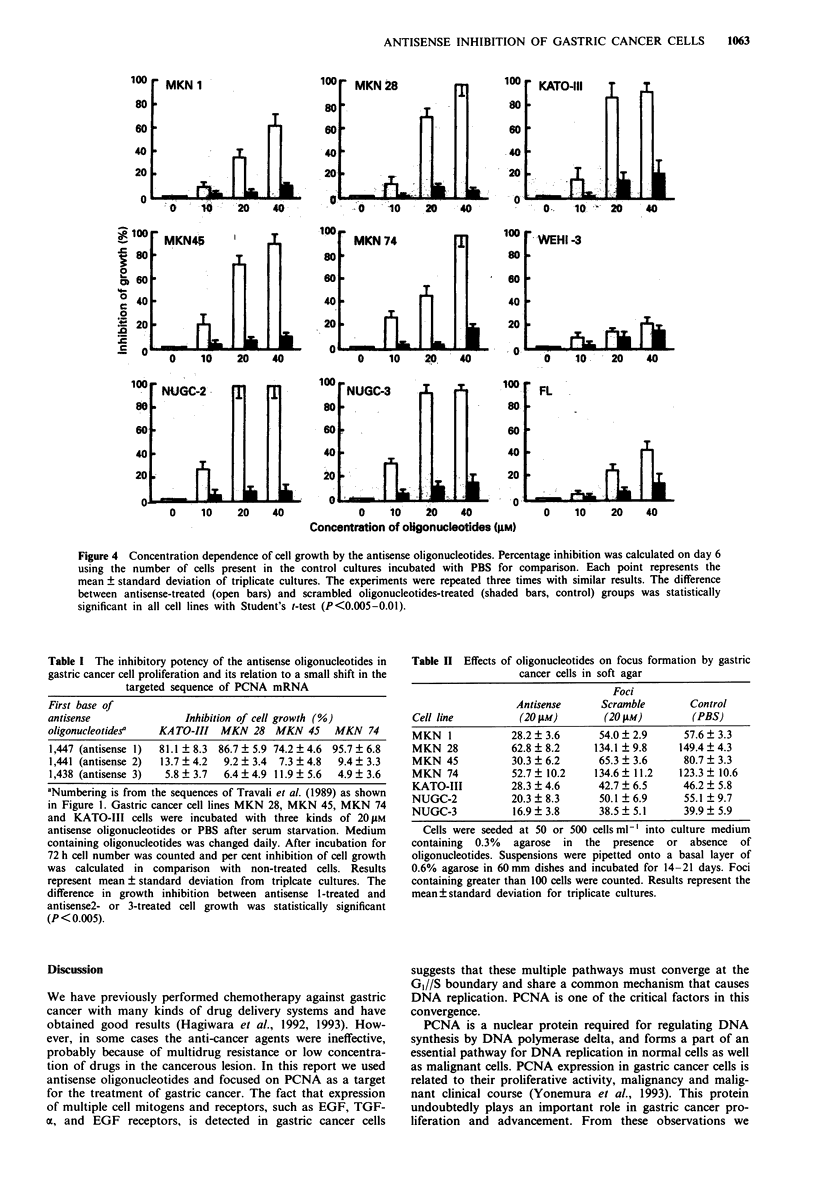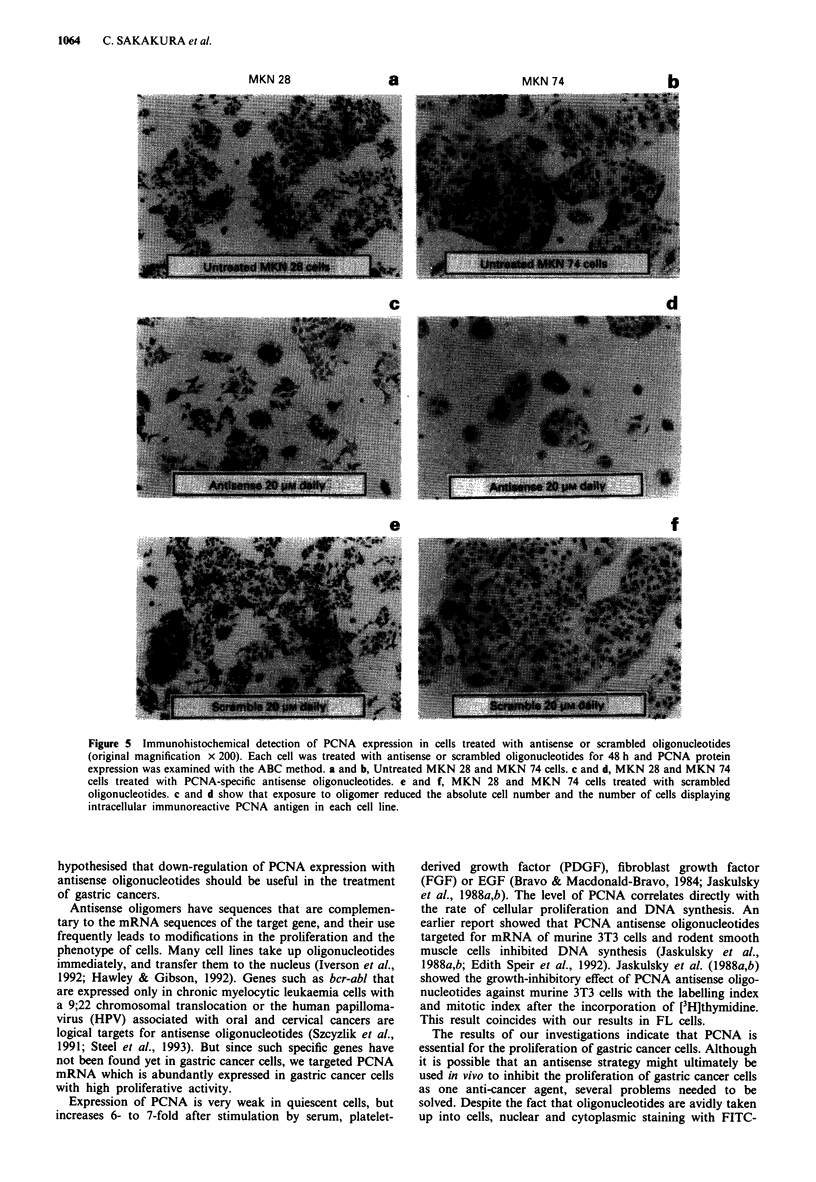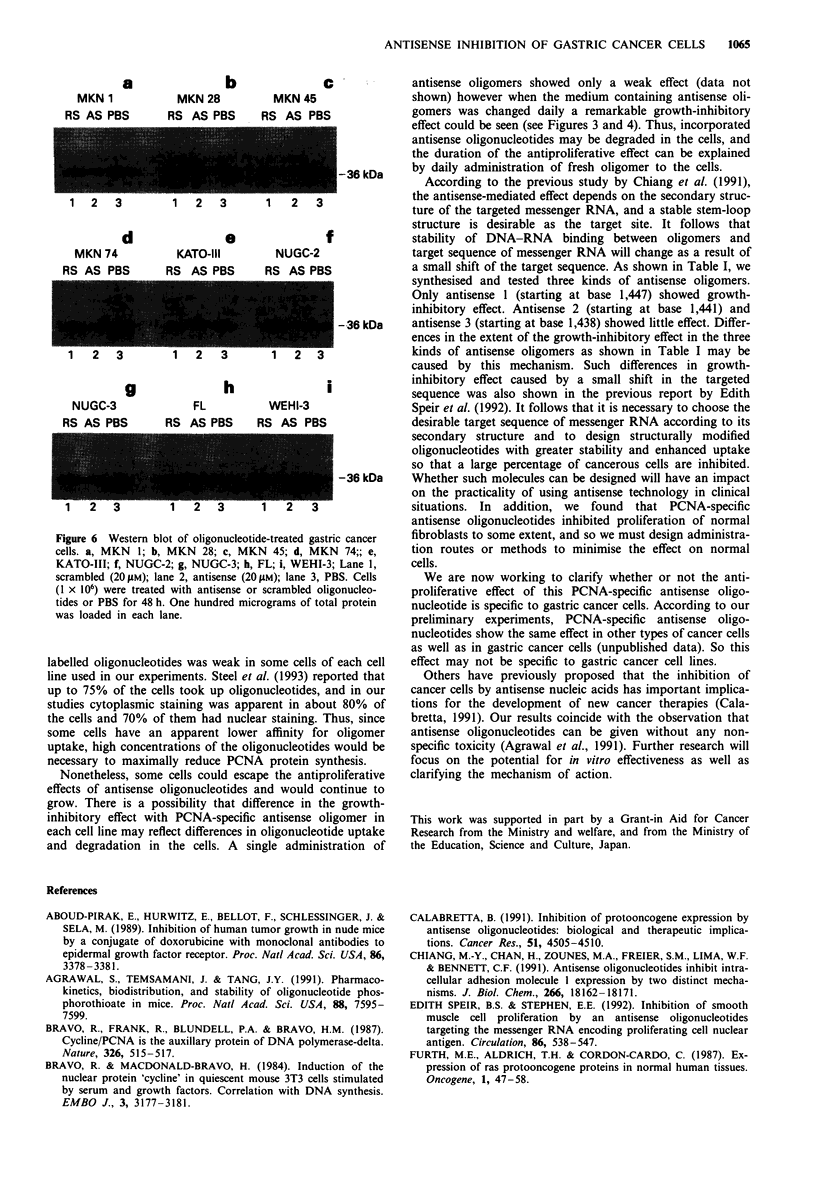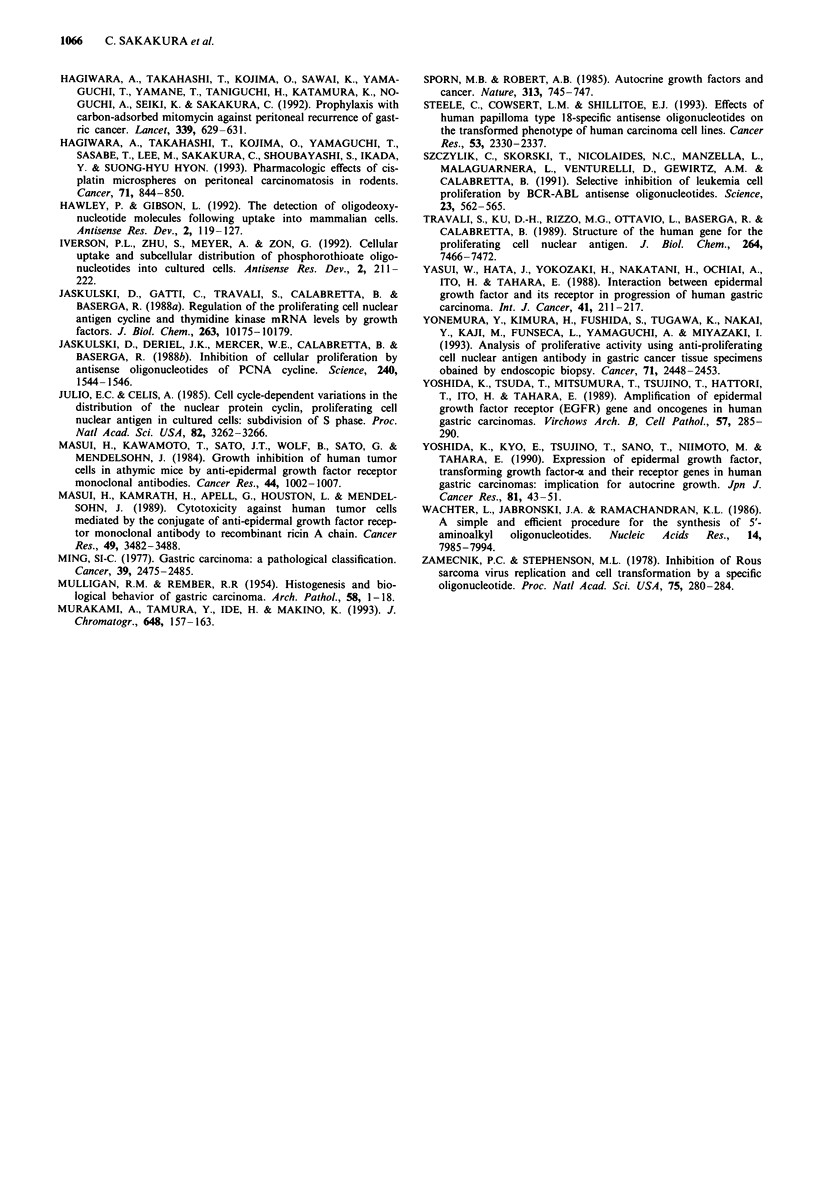# Inhibition of gastric cancer cell proliferation by antisense oligonucleotides targeting the messenger RNA encoding proliferating cell nuclear antigen.

**DOI:** 10.1038/bjc.1994.449

**Published:** 1994-12

**Authors:** C. Sakakura, A. Hagiwara, H. Tsujimoto, K. Ozaki, T. Sakakibara, T. Oyama, M. Ogaki, T. Takahashi

**Affiliations:** First Department of Surgery, Kyoto Prefectural University of Medicine, Japan.

## Abstract

**Images:**


Br. J. Cancer (1994), 70, 1060-1066 © Macmillan Press Ltd., 1994

### Inhibition of gastric cancer cell proliferation by antisense oligonucleotides targeting the messenger RNA encoding proliferating cell nuclear antigen*

C. Sakakura[1], A. Hagiwara[1], H. Tsujimoto[1], K. Ozaki[1], T. Sakakibara[1], T. Oyama[1], M. Ogaki[1] & T. Takahashi[1]

[1]*First Department of Surgery, Kyoto Prefectural University of Medicine, Kamigyo-ku, Kawaramachi-dori, Kyoto 602 Japan.*

**Summary** Proliferating cell nuclear antigen (PCNA) is a nuclear protein that regulates DNA synthesis by DNA polymerase delta, and is essential for DNA replication. PCNA expression level is related to the malignancy of gastric cancer cells. Seven different gastric cancer cell lines and two kinds of control cell lines were treated with antisense oligonucleotides complementary to the messenger RNA of PCNA. Treatment of each gastric cancer cell line with antisense oligonucleotides at concentration of 10-40 μM inhibited the cell growth, colony formation and PCNA protein production in a dose-dependent manner, but only affected normal cells slightly. A random sequence oligomer showed no effect. These results show that PCNA is essential for gastric cancer cell proliferation and that the use of synthetic oligonucleotides is an effective way of producing antisense-mediated changes in the behaviour of human gastric cancers.

Gastric cancer is morphologically and functionally pleomorphic (Mulligan & Rember, 1958; Ming, 1977), and it has been suggested that many kinds of growth factors and their receptors form multiautocrine loops that regulate cancer cell growth and development (Yoshida *et al.*, 1989). Expression of epidermal growth factor (EGF), transforming growth factor α (TGF-α), EGF receptor and PCNA is apparently involved in the malignancy of gastric cancer cells (Yasui *et al.*, 1988; Yoshida *et al.*, 1990; Yonemura *et al.*, 1993).

Recent investigations have shown that oncogenes encode growth factors, their receptors and other parts of the signal transduction mechanisms, all of which play important roles in controlling the growth of cancerous and normal cells (Sporn & Roberts, 1985). The signal transduction mechanisms often involve a final common pathway that is shared by diverse growth signals. The convergence to a common pathway is illustrated by a nuclear protein, proliferating cell nuclear antigen (PCNA), which is critical for DNA replication. PCNA is a nuclear protein required for regulating DNA synthesis by DNA polymerase delta, which plays an essential role in DNA replication (Julio & Celis, 1985; Bravo *et al.*, 1987). PCNA constitutes an important factor in this process; it is a cofactor for DNA polymerase delta, and both the cofactor and the enzyme are required for coordinated leading and lagging strand replication of DNA.

One of the general approaches previously explored for the treatment of cancer was the development of interventions that inhibit specific factors involved in the signal transduction pathways leading to cell division (Masui *et al.*, 1984; Aboud-Pirak *et al.*, 1989; Masui *et al.*, 1989). Nonetheless, if one such pathway is inhibited, other pathways might still produce substantial cell proliferation. Consequently, to effectively suppress cell proliferation the intracellular factors that are involved in a final common pathway, shared by mitogenic signals, should be targeted. PCNA is one of the intracellular factors that is common to all pathways of DNA synthesis.

Since gastric cancers with high PCNA expression show a more malignant clinical course than those with low PCNA expression (Yonemura *et al.*, 1993), it follows that inhibition of PCNA expression in gastric cancer cells should reduce their malignancy and improve the clinical course. The present study was performed to determine whether antisense oligonucleotides complementary to the messenger RNA (mRNA) of PCNA would inhibit PCNA expression and thereby inhibit the proliferation of gastric cancer cells. If proliferation of gastric cancer cells could be inhibited by antisense oligonucleotides specific to PCNA mRNA, their application might be a useful chemotherapeutic strategy for treating cancer.

Correspondence: C. Sakakura, MD. The Biomembrane Institute, 201 Elliott Avenue, Suite 305, Seattle, Washington, USA.
Received 2 June 1994; and in revised form 26 July 1994.

### Materials and methods

### *Cell culture*

The human gastric cancer cell lines MKN1, MKN28, MKN45, MKN74, NUGC-2 and NUGC-3 were cultured in RPMI-1640 (Nissui, Tokyo) with 10% fetal bovine serum (FBS) and 100 IU of penicillin at 37°C under standard conditions.

The human myelomonocytic cell line WEHI-3 and human fibroblast FL cells were cultured in the same conditions and used as control cells. Another gastric cancer cell line, KATO-III, was cultured in Dulbecco's modified Eagle medium supplemented with 10% FBS.

### *Oligonucleotide synthesis*

Eighteen-base oligonucleotides were synthesised using the Applied Biosystems 380B DNA synthesiser (Applied Biosystems, Foster City, CA, USA) with a phosphorothioate substitution at each base. The oligomers were purified by two different high-performance liquid chromatography (HPLC) methods (Murakami *et al.*, 1993), and purity was assayed by polyacrylamide gel electrophoresis and HPLC. The antisense oligonucleotides were complementary to 18-bp sequences next to the start codon or overlapping the start codon of PCNA mRNA sequences, as shown in Figure 1. Sterile aliquots of 1 mM stock solutions were stored at -20°C and thawed on ice before use.

### *Oligonucleotide uptake*

Random sequence phosphorothioate oligonucleotides were conjugated with fluorescein 5-isothiocyanate (Fluorescein-ON Phosphoramidite; Clontech, Palo Alto, CA, USA) according to the procedure of Wachter *et al.* (1986). Cells were seeded at a density of $5 \times 10^3$ ml$^{-1}$ in 60 mm tissue culture dishes. After 24 h, the media were changed, and three 10 mm glass cloning cylinders were plated on each plate. The labelled oligonucleotides were then added at a concentration of 5 μM

to the medium within the cloning cylinders. Plates were harvested at 30 min, 1 h and then hourly. The cells were washed several times in cold phosphate buffer (PBS), and fixed for 5 min in 10% formaldehyde. After fixation, the plates were washed again with PBS, and coverslips were mounted using Vectashield (Vector Laboratories, Burlingame, CA, USA). Cultures were viewed and photographed immediately under a UV fluorescent microscope.

*Growth rate*

Cells were plated at a density of $1 \times 10^4$ cells $ml^{-1}$ into 24-well dishes for 24 h. The medium was then changed to one containing 10% FBS and various concentrations (10, 20 or 40 μM) of either antisense oligomer, scrambled oligomer or PBS were added. Daily, the cells were digested with trypsin and counted. Each test was performed in triplicate and repeated at least three times for each concentration of oligonucleotides. The growth medium, with or without oligonucleotides was changed daily.

**Figure 1** The sequences of PCNA mRNA targeted by the antisense oligonucleotides. Three targeted sequences are boxed with corresponding nucleotide number from sequences of Travali *et al.* (1989). The start codon of the gene is underlined. The inhibitory potency of the antisense oligonucleotides was related to the small shift in the sequence targeted. *Inhibits gastric cancer cell proliferation; **No effect.

*Anchorage independence*

Anchorage independence was assayed by seeding cells in 0.3% SeaKem low melting point agarose (FMC Bioproducts, Rockland, ME, USA) dissolved in 2 × RPMI media with or without oligonucleotides. Suspensions, containing 50 or 500 cells $ml^{-1}$, were overlaid on a 0.6% agarose basal layer in 60 mm culture dishes and incubated at 37°C for 14–21 days. Foci containing more than 100 cells counted.

*Immunohistochemical detection of PCNA*

Cells were seeded at 5,000 $cm^{-2}$ in four-well chamber slides for 24 h culture, new medium containing 10% FBS and either 20 μM antisense or scrambled oligonucleotides was added and the slides were incubated for 48 h. The cells were rinsed with PBS and the percentage PCNA expression was detected using the avidin–biotin peroxidase complex method (Furth *et al.*, 1987) with the VECTASTAIN ABC kit (Vector Laboratory, Burlingame CA, USA). All subsequent procedures were performed at room temperature. Non-specific binding sites were blocked with 10% normal horse serum for 30 min. The serum was removed and the cells were then incubated with 10 μM monoclonal anti-PCNA antibody (Dako-PCNA, PC10 Glostrup, Denmark) for 1 h. After rinsing with PBS for 15 min, the slides were incubated with peroxidase-conjugated goat anti-mouse IgG + IgM (Jackson

**Figure 2** Uptake of random sequence oligonucleotides in MKN 74 cells, as seen by UV fluorescence microscopy. Fluoresceinated oligonucleotides were added to the culture, which was then photographed at ×400 after 30 min (**a**), 1 h (**b**), 2 h (**c**) and 4 h (**d**).

ImmunoResearch Laboratory, PA, USA) for 30 min. Following a 15 min PBS rinse, the cells were incubated for 30 min with avidin DH-biotinylated horseradish peroxidase H complex (ABC). The slides were rinsed for 10 min and reacted with diaminobenzidine in 0.01% hydrogen peroxide solution for 5 min. After a final PBS rinse the samples were counterstained with haematoxylin, dehydrated in ethanol, cleared in xylene and mounted under coverslips using Permount.

*Western blot analysis*

Cells were cultured in complete medium containing 20 μM oligonucleotides for 48 h. Cells were trypsinised, flash-frozen in liquid nitrogen and incubated in lysis buffer [0.01 M Tris-HCl (pH 7.5), 0.144 M sodium chloride, 0.5% NP-40, 0.5% SDS and 0.1% aprotinin ($1 \times 10^6$ cells per 20 μl of buffer)] for 30 min on ice and then vortexed. The lysates were centrifuged at 10,000 *g* for 10 min and subjected to 6-12% SDS-PAGE at 20 mA for 2.5 h. Each well was loaded with approximately 20 μl (100 μg of protein) of the samples. The proteins were transferred for 90 min at 100 V in a Polyblot (American Bionetics, Hayward, CA, USA). Immunoblot was performed using anti-PCNA monoclonal antibody at dilution of 1:1,000, and then with peroxidase-conjugated secondary antibody at a dilution of 1:5,000.

*Statistical analysis*

Differences in inhibition of cell growth and colony formation were evaluated with Student's *t*-test.

**Figure 3** Growth curves of gastric cancer cells and control cells. MKN 28 (**a**), MKN 74 (**b**), KATO-III (**c**) and FL (**d**) cells were incubated with 20 μM antisense (▲) or sense (○) oligonucleotides or phosphate-buffered saline (●). Cells were seeded in growth medium on day 0, and incubated for 24 h. The cells were then incubated with 20 μM antisense or sense oligonucleotides or phosphate-buffered saline in fresh medium. Medium containing oligonucleotides was changed daily. The experiments were repeated three times. Results represent mean ± standard deviation from triplicate cultures. The difference between antisense-treated and control curves was statistically significant with Student's *t*-test ($P<0.01$).

### Results

*Oligonucleotide uptake*

Fluorescence microscopy revealed that all cells were capable of taking up the oligonucleotides. After 30 min of exposure, faint fluorescence was seen on the outer membrane, and by 1 h about 80% of the cells showed diffuse cytoplasmic staining. Within 2 h, up to 70% of the cells showed very intense nuclear staining. By 4 h, the nuclear fluorescence had disappeared to be replaced by a coarse granular staining of the cytoplasm, and therefore no fluorescence was detectable. All cell types showed a similar time course and pattern of staining (Figure 2).

*Growth rate*

When plated at an initial density of $1 \times 10^4$ cells ml$^{-1}$, all cell lines grew to maximum density within 6 days. At all times examined the growth of all gastric cancer cell lines was inhibited by the presence of the antisense oligonucleotides, but to a lesser degree in FL cells and WEHI-3 cells. Random sequence oligonucleotides showed no effect on the growth of any cell lines. The growth inhibition of MKN28, MKN74 and KATO-III cells is shown in Figure 3. The other gastric cancer cells showed similar results. Treatment with 10-40 μM antisense oligonucleotides inhibited the growth of gastric cancer cells in a dose-dependent manner, but each oligomer showed only a slight effect on the proliferation of FL cells and WEHI-3 cells (Figures 3 and 4).

When the composition of the antisense oligonucleotides was altered to make them complementary to a slightly 3′ region that spanned the start codon, significant differences were observed in the growth-inhibitory effect. The inhibitory effect of three kinds of antisense oligonucleotides against MKN28, MKN45, MKN74 and KATO-III cells was compared. Antisense oligonucleotides starting at base 1,447 showed growth inhibition of each cell line, but the other two antisense oligonucleotides starting at base 1441 or starting at base 1,438 did not show inhibition of cell proliferation (Table I).

*Anchorage independence*

The ability to form foci in soft agar was reduced by antisense oligonucleotide treatment in each gastric cancer cell line (Table II).

*Effect of PCNA-specific antisense oligonucleotides on protein expression: immunohistochemical analysis*

In growing gastric cancer cells without treatment or treated with 20 μM scrambled oligonucleotides for 48 h, most nuclei were stained with similar high intensities. In contrast, more than 70% of gastric cancer cells treated with 20 μM antisense oligonucleotides for 48 h showed weakly stained nuclei. Figure 5a and b show untreated MKN28 and MKN74 cells, Figure 5c and d MKN28 and MKN74 cells treated with PCNA-specific antisense oligomer and Figure 5e and f MKN28 and MKN74 cells exposed to scrambled oligonucleotides. The results presented in Figure 5c and d show that exposure to antisense oligomers reduced the absolute cell number and the number of cells displaying intranuclear immunoreactive PCNA antigen in each cell line. Other cell lines showed similar results.

*Effect of PCNA-specific antisense oligonucleotides on protein expression: Western blots*

Immunoblots demonstrated a marked decrease in the level of PCNA protein after incubation in antisense oligomer, whereas no decrease was apparent in the cells exposed to scrambled oligonucleotides or PBS in each kind of gastric cancer cells (Figure 6a-g). The PCNA protein level in normal cells, FL cells and WEHI-3 cells also decreased slightly (Figure 6h and i).

**Figure 4** Concentration dependence of cell growth by the antisense oligonucleotides. Percentage inhibition was calculated on day 6 using the number of cells present in the control cultures incubated with PBS for comparison. Each point represents the mean ± standard deviation of triplicate cultures. The experiments were repeated three times with similar results. The difference between antisense-treated (open bars) and scrambled oligonucleotides-treated (shaded bars, control) groups was statistically significant in all cell lines with Student's *t*-test ($P<0.005-0.01$).

**Table I** The inhibitory potency of the antisense oligonucleotides in gastric cancer cell proliferation and its relation to a small shift in the targeted sequence of PCNA mRNA

| *First base of antisense oligonucleotides*[a] | *Inhibition of cell growth (%)* KATO-III | MKN 28 | MKN 45 | MKN 74 |
|---|---|---|---|---|
| 1,447 (antisense 1) | 81.1 ± 8.3 | 86.7 ± 5.9 | 74.2 ± 4.6 | 95.7 ± 6.8 |
| 1,441 (antisense 2) | 13.7 ± 4.2 | 9.2 ± 3.4 | 7.3 ± 4.8 | 9.4 ± 3.3 |
| 1,438 (antisense 3) | 5.8 ± 3.7 | 6.4 ± 4.9 | 11.9 ± 5.6 | 4.9 ± 3.6 |

[a]Numbering is from the sequences of Travali *et al.* (1989) as shown in Figure 1. Gastric cancer cell lines MKN 28, MKN 45, MKN 74 and KATO-III cells were incubated with three kinds of 20 μM antisense oligonucleotides or PBS after serum starvation. Medium containing oligonucleotides was changed daily. After incubation for 72 h cell number was counted and per cent inhibition of cell growth was calculated in comparison with non-treated cells. Results represent mean ± standard deviation from triplcate cultures. The difference in growth inhibition between antisense 1-treated and antisense2- or 3-treated cell growth was statistically significant ($P<0.005$).

**Table II** Effects of oligonucleotides on focus formation by gastric cancer cells in soft agar

| *Cell line* | *Antisense (20 μM)* | *Foci Scramble (20 μM)* | *Control (PBS)* |
|---|---|---|---|
| MKN 1 | 28.2 ± 3.6 | 54.0 ± 2.9 | 57.6 ± 3.3 |
| MKN 28 | 62.8 ± 8.2 | 134.1 ± 9.8 | 149.4 ± 4.3 |
| MKN 45 | 30.3 ± 6.2 | 65.3 ± 3.6 | 80.7 ± 3.3 |
| MKN 74 | 52.7 ± 10.2 | 134.6 ± 11.2 | 123.3 ± 10.6 |
| KATO-III | 28.3 ± 4.6 | 42.7 ± 6.5 | 46.2 ± 5.8 |
| NUGC-2 | 20.3 ± 8.3 | 50.1 ± 6.9 | 55.1 ± 9.7 |
| NUGC-3 | 16.9 ± 3.8 | 38.5 ± 5.1 | 39.9 ± 5.9 |

Cells were seeded at 50 or 500 cells $ml^{-1}$ into culture medium containing 0.3% agarose in the presence or absence of oligonucleotides. Suspensions were pipetted onto a basal layer of 0.6% agarose in 60 mm dishes and incubated for 14–21 days. Foci containing greater than 100 cells were counted. Results represent the mean ± standard deviation for triplicate cultures.

### Discussion

We have previously performed chemotherapy against gastric cancer with many kinds of drug delivery systems and have obtained good results (Hagiwara *et al.*, 1992, 1993). However, in some cases the anti-cancer agents were ineffective, probably because of multidrug resistance or low concentration of drugs in the cancerous lesion. In this report we used antisense oligonucleotides and focused on PCNA as a target for the treatment of gastric cancer. The fact that expression of multiple cell mitogens and receptors, such as EGF, TGF-α, and EGF receptors, is detected in gastric cancer cells suggests that these multiple pathways must converge at the $G_1//S$ boundary and share a common mechanism that causes DNA replication. PCNA is one of the critical factors in this convergence.

PCNA is a nuclear protein required for regulating DNA synthesis by DNA polymerase delta, and forms a part of an essential pathway for DNA replication in normal cells as well as malignant cells. PCNA expression in gastric cancer cells is related to their proliferative activity, malignancy and malignant clinical course (Yonemura *et al.*, 1993). This protein undoubtedly plays an important role in gastric cancer proliferation and advancement. From these observations we

**Figure 5** Immunohistochemical detection of PCNA expression in cells treated with antisense or scrambled oligonucleotides (original magnification × 200). Each cell was treated with antisense or scrambled oligonucleotides for 48 h and PCNA protein expression was examined with the ABC method. **a** and **b**, Untreated MKN 28 and MKN 74 cells. **c** and **d**, MKN 28 and MKN 74 cells treated with PCNA-specific antisense oligonucleotides. **e** and **f**, MKN 28 and MKN 74 cells treated with scrambled oligonucleotides. **c** and **d** show that exposure to oligomer reduced the absolute cell number and the number of cells displaying intracellular immunoreactive PCNA antigen in each cell line.

hypothesised that down-regulation of PCNA expression with antisense oligonucleotides should be useful in the treatment of gastric cancers.

Antisense oligomers have sequences that are complementary to the mRNA sequences of the target gene, and their use frequently leads to modifications in the proliferation and the phenotype of cells. Many cell lines take up oligonucleotides immediately, and transfer them to the nucleus (Iverson *et al.*, 1992; Hawley & Gibson, 1992). Genes such as *bcr-abl* that are expressed only in chronic myelocytic leukaemia cells with a 9;22 chromosomal translocation or the human papillomavirus (HPV) associated with oral and cervical cancers are logical targets for antisense oligonucleotides (Szcyzlik *et al.*, 1991; Steel *et al.*, 1993). But since such specific genes have not been found yet in gastric cancer cells, we targeted PCNA mRNA which is abundantly expressed in gastric cancer cells with high proliferative activity.

Expression of PCNA is very weak in quiescent cells, but increases 6- to 7-fold after stimulation by serum, platelet-derived growth factor (PDGF), fibroblast growth factor (FGF) or EGF (Bravo & Macdonald-Bravo, 1984; Jaskulsky *et al.*, 1988*a*,*b*). The level of PCNA correlates directly with the rate of cellular proliferation and DNA synthesis. An earlier report showed that PCNA antisense oligonucleotides targeted for mRNA of murine 3T3 cells and rodent smooth muscle cells inhibited DNA synthesis (Jaskulsky *et al.*, 1988*a*,*b*; Edith Speir *et al.*, 1992). Jaskulsky *et al.* (1988*a*,*b*) showed the growth-inhibitory effect of PCNA antisense oligonucleotides against murine 3T3 cells with the labelling index and mitotic index after the incorporation of [$^3$H]thymidine. This result coincides with our results in FL cells.

The results of our investigations indicate that PCNA is essential for the proliferation of gastric cancer cells. Although it is possible that an antisense strategy might ultimately be used *in vivo* to inhibit the proliferation of gastric cancer cells as one anti-cancer agent, several problems needed to be solved. Despite the fact that oligonucleotides are avidly taken up into cells, nuclear and cytoplasmic staining with FITC-

**Figure 6** Western blot of oligonucleotide-treated gastric cancer cells. **a**, MKN 1; **b**, MKN 28; **c**, MKN 45; **d**, MKN 74;; **e**, KATO-III; **f**, NUGC-2; **g**, NUGC-3; **h**, FL; **i**, WEHI-3; Lane 1, scrambled (20 µM); lane 2, antisense (20 µM); lane 3, PBS. Cells ($1 \times 10^6$) were treated with antisense or scrambled oligonucleotides or PBS for 48 h. One hundred micrograms of total protein was loaded in each lane.

labelled oligonucleotides was weak in some cells of each cell line used in our experiments. Steel *et al.* (1993) reported that up to 75% of the cells took up oligonucleotides, and in our studies cytoplasmic staining was apparent in about 80% of the cells and 70% of them had nuclear staining. Thus, since some cells have an apparent lower affinity for oligomer uptake, high concentrations of the oligonucleotides would be necessary to maximally reduce PCNA protein synthesis.

Nonetheless, some cells could escape the antiproliferative effects of antisense oligonucleotides and would continue to grow. There is a possibility that difference in the growth-inhibitory effect with PCNA-specific antisense oligomer in each cell line may reflect differences in oligonucleotide uptake and degradation in the cells. A single administration of antisense oligomers showed only a weak effect (data not shown) however when the medium containing antisense oligomers was changed daily a remarkable growth-inhibitory effect could be seen (see Figures 3 and 4). Thus, incorporated antisense oligonucleotides may be degraded in the cells, and the duration of the antiproliferative effect can be explained by daily administration of fresh oligomer to the cells.

According to the previous study by Chiang *et al.* (1991), the antisense-mediated effect depends on the secondary structure of the targeted messenger RNA, and a stable stem-loop structure is desirable as the target site. It follows that stability of DNA–RNA binding between oligomers and target sequence of messenger RNA will change as a result of a small shift of the target sequence. As shown in Table I, we synthesised and tested three kinds of antisense oligomers. Only antisense 1 (starting at base 1,447) showed growth-inhibitory effect. Antisense 2 (starting at base 1,441) and antisense 3 (starting at base 1,438) showed little effect. Differences in the extent of the growth-inhibitory effect in the three kinds of antisense oligomers as shown in Table I may be caused by this mechanism. Such differences in growth-inhibitory effect caused by a small shift in the targeted sequence was also shown in the previous report by Edith Speir *et al.* (1992). It follows that it is necessary to choose the desirable target sequence of messenger RNA according to its secondary structure and to design structurally modified oligonucleotides with greater stability and enhanced uptake so that a large percentage of cancerous cells are inhibited. Whether such molecules can be designed will have an impact on the practicality of using antisense technology in clinical situations. In addition, we found that PCNA-specific antisense oligonucleotides inhibited proliferation of normal fibroblasts to some extent, and so we must design administration routes or methods to minimise the effect on normal cells.

We are now working to clarify whether or not the antiproliferative effect of this PCNA-specific antisense oligonucleotide is specific to gastric cancer cells. According to our preliminary experiments, PCNA-specific antisense oligonucleotides show the same effect in other types of cancer cells as well as in gastric cancer cells (unpublished data). So this effect may not be specific to gastric cancer cell lines.

Others have previously proposed that the inhibition of cancer cells by antisense nucleic acids has important implications for the development of new cancer therapies (Calabretta, 1991). Our results coincide with the observation that antisense oligonucleotides can be given without any non-specific toxicity (Agrawal *et al.*, 1991). Further research will focus on the potential for *in vitro* effectiveness as well as clarifying the mechanism of action.

This work was supported in part by a Grant-in Aid for Cancer Research from the Ministry and welfare, and from the Ministry of the Education, Science and Culture, Japan.

### References

ABOUD-PIRAK, E., HURWITZ, E., BELLOT, F., SCHLESSINGER, J. & SELA, M. (1989). Inhibition of human tumor growth in nude mice by a conjugate of doxorubicine with monoclonal antibodies to epidermal growth factor receptor. *Proc. Natl Acad. Sci. USA*, **86**, 3378–3381.

AGRAWAL, S., TEMSAMANI, J. & TANG, J.Y. (1991). Pharmacokinetics, biodistribution, and stability of oligonucleotide phosphorothioate in mice. *Proc. Natl Acad. Sci. USA*, **88**, 7595–7599.

BRAVO, R., FRANK, R., BLUNDELL, P.A. & BRAVO, H.M. (1987). Cycline/PCNA is the auxillary protein of DNA polymerase-delta. *Nature*, **326**, 515–517.

BRAVO, R. & MACDONALD-BRAVO, H. (1984). Induction of the nuclear protein 'cycline' in quiescent mouse 3T3 cells stimulated by serum and growth factors. Correlation with DNA synthesis. *EMBO J.*, **3**, 3177–3181.

CALABRETTA, B. (1991). Inhibition of protooncogene expression by antisense oligonucleotides: biological and therapeutic implications. *Cancer Res.*, **51**, 4505–4510.

CHIANG, M.-Y., CHAN, H., ZOUNES, M.A., FREIER, S.M., LIMA, W.F. & BENNETT, C.F. (1991). Antisense oligonucleotides inhibit intracellular adhesion molecule 1 expression by two distinct mechanisms. *J. Biol. Chem.*, **266**, 18162–18171.

EDITH SPEIR, B.S. & STEPHEN, E.E. (1992). Inhibition of smooth muscle cell proliferation by an antisense oligonucleotides targeting the messenger RNA encoding proliferating cell nuclear antigen. *Circulation*, **86**, 538–547.

FURTH, M.E., ALDRICH, T.H. & CORDON-CARDO, C. (1987). Expression of ras protooncogene proteins in normal human tissues. *Oncogene*, **1**, 47–58.

HAGIWARA, A., TAKAHASHI, T., KOJIMA, O., SAWAI, K., YAMAGUCHI, T., YAMANE, T., TANIGUCHI, H., KATAMURA, K., NOGUCHI, A., SEIKI, K. & SAKAKURA, C. (1992). Prophylaxis with carbon-adsorbed mitomycin against peritoneal recurrence of gastric cancer. *Lancet*, **339**, 629–631.

HAGIWARA, A., TAKAHASHI, T., KOJIMA, O., YAMAGUCHI, T., SASABE, T., LEE, M., SAKAKURA, C., SHOUBAYASHI, S., IKADA, Y. & SUONG-HYU HYON. (1993). Pharmacologic effects of cisplatin microspheres on peritoneal carcinomatosis in rodents. *Cancer*, **71**, 844–850.

HAWLEY, P. & GIBSON, L. (1992). The detection of oligodeoxynucleotide molecules following uptake into mammalian cells. *Antisense Res. Dev.*, **2**, 119–127.

IVERSON, P.L., ZHU, S., MEYER, A. & ZON, G. (1992). Cellular uptake and subcellular distribution of phosphorothioate oligonucleotides into cultured cells. *Antisense Res. Dev.*, **2**, 211–222.

JASKULSKI, D., GATTI, C., TRAVALI, S., CALABRETTA, B. & BASERGA, R. (1988*a*). Regulation of the proliferating cell nuclear antigen cycline and thymidine kinase mRNA levels by growth factors. *J. Biol. Chem.*, **263**, 10175–10179.

JASKULSKI, D., DERIEL, J.K., MERCER, W.E., CALABRETTA, B. & BASERGA, R. (1988*b*). Inhibition of cellular proliferation by antisense oligonucleotides of PCNA cycline. *Science*, **240**, 1544–1546.

JULIO, E.C. & CELIS, A. (1985). Cell cycle-dependent variations in the distribution of the nuclear protein cyclin, proliferating cell nuclear antigen in cultured cells: subdivision of S phase. *Proc. Natl Acad. Sci. USA*, **82**, 3262–3266.

MASUI, H., KAWAMOTO, T., SATO, J.T., WOLF, B., SATO, G. & MENDELSOHN, J. (1984). Growth inhibition of human tumor cells in athymic mice by anti-epidermal growth factor receptor monoclonal antibodies. *Cancer Res.*, **44**, 1002–1007.

MASUI, H., KAMRATH, H., APELL, G., HOUSTON, L. & MENDELSOHN, J. (1989). Cytotoxicity against human tumor cells mediated by the conjugate of anti-epidermal growth factor receptor monoclonal antibody to recombinant ricin A chain. *Cancer Res.*, **49**, 3482–3488.

MING, SI-C. (1977). Gastric carcinoma: a pathological classification. *Cancer*, **39**, 2475–2485.

MULLIGAN, R.M. & REMBER, R.R (1954). Histogenesis and biological behavior of gastric carcinoma. *Arch. Pathol.*, **58**, 1–18.

MURAKAMI, A., TAMURA, Y., IDE, H. & MAKINO, K. (1993). *J. Chromatogr.*, **648**, 157–163.

SPORN, M.B. & ROBERT, A.B. (1985). Autocrine growth factors and cancer. *Nature*, **313**, 745–747.

STEELE, C., COWSERT, L.M. & SHILLITOE, E.J. (1993). Effects of human papilloma type 18-specific antisense oligonucleotides on the transformed phenotype of human carcinoma cell lines. *Cancer Res.*, **53**, 2330–2337.

SZCZYLIK, C., SKORSKI, T., NICOLAIDES, N.C., MANZELLA, L., MALAGUARNERA, L., VENTURELLI, D., GEWIRTZ, A.M. & CALABRETTA, B. (1991). Selective inhibition of leukemia cell proliferation by BCR-ABL antisense oligonucleotides. *Science*, **23**, 562–565.

TRAVALI, S., KU, D.-H., RIZZO, M.G., OTTAVIO, L., BASERGA, R. & CALABRETTA, B. (1989). Structure of the human gene for the proliferating cell nuclear antigen. *J. Biol. Chem.*, **264**, 7466–7472.

YASUI, W., HATA, J., YOKOZAKI, H., NAKATANI, H., OCHIAI, A., ITO, H. & TAHARA, E. (1988). Interaction between epidermal growth factor and its receptor in progression of human gastric carcinoma. *Int. J. Cancer*, **41**, 211–217.

YONEMURA, Y., KIMURA, H., FUSHIDA, S., TUGAWA, K., NAKAI, Y., KAJI, M., FUNSECA, L., YAMAGUCHI, A. & MIYAZAKI, I. (1993). Analysis of proliferative activity using anti-proliferating cell nuclear antigen antibody in gastric cancer tissue specimens obained by endoscopic biopsy. *Cancer*, **71**, 2448–2453.

YOSHIDA, K., TSUDA, T., MITSUMURA, T., TSUJINO, T., HATTORI, T., ITO, H. & TAHARA, E. (1989). Amplification of epidermal growth factor receptor (EGFR) gene and oncogenes in human gastric carcinomas. *Virchows Arch. B, Cell Pathol.*, **57**, 285–290.

YOSHIDA, K., KYO, E., TSUJINO, T., SANO, T., NIIMOTO, M. & TAHARA, E. (1990). Expression of epidermal growth factor, transforming growth factor-α and their receptor genes in human gastric carcinomas: implication for autocrine growth. *Jpn J. Cancer Res.*, **81**, 43–51.

WACHTER, L., JABRONSKI, J.A. & RAMACHANDRAN, K.L. (1986). A simple and efficient procedure for the synthesis of 5′-aminoalkyl oligonucleotides. *Nucleic Acids Res.*, **14**, 7985–7994.

ZAMECNIK, P.C. & STEPHENSON, M.L. (1978). Inhibition of Rous sarcoma virus replication and cell transformation by a specific oligonucleotide. *Proc. Natl Acad. Sci. USA*, **75**, 280–284.